# Research on Laser Cleaning Technology for Aircraft Skin Surface Paint Layer

**DOI:** 10.3390/ma17102414

**Published:** 2024-05-17

**Authors:** Jinxuan Li, Jianjun Yang, Jiaxuan Liu, Hui Chen, Yunfei Duan, Xinjian Pan

**Affiliations:** 1 College of Electron and Information, University of Electronic Science and Technology of China, Zhongshan Institute, Zhongshan 528402, China; leejinxuanya@163.com (J.L.); liujiaxuan906@163.com (J.L.); 17752332766@163.com (H.C.); xinjpan@163.com (X.P.); 2 College of Physics and Optoelectronic Engineering, Shenzhen University, Shenzhen 518060, China; dkness121@163.com

**Keywords:** laser cleaning, microstructure evolution, cleaning mechanisms, thermal ablation, thermal vibration

## Abstract

In this study, a pulsed laser operating at a wavelength of 1064 nm and with a pulse width of 100 ns was utilized for the removal of paint from the surface of a 2024 aluminum alloy. The experimental investigation was conducted to analyze the influence of laser parameters on the efficacy of paint layer removal from the aircraft skin’s surface and the subsequent evolution in the microstructure of the laser-treated aluminum alloy substrate. The mechanism underlying laser cleaning was explored through simulation. The findings revealed that power density and scanning speed significantly affected the quality of cleaning. Notably, there were discernible damage thresholds and optimal cleaning parameters in repetitive frequency, with a power density of 178.25 MW/cm^2^, scanning speed of 500 mm/s, and repetitive frequency of 40 kHz identified as the primary optimal settings for achieving the desired cleaning effect. Thermal ablation and thermal vibration were identified as the principal mechanisms of cleaning. Moreover, laser processing induced surface dislocations and concentrated stress, accompanied by grain refinement, on the aluminum substrate.

## 1. Introduction

Aircraft skins are commonly manufactured using aluminum alloy due to its superior strength, durability, and resistance to corrosion [1]. The surface paint layer of an aircraft will age, crack, peel, and sustain other problems due to airflow wash, vibration fatigue, and other factors [2,3]; consequently, it is essential to clean the paint layer to restore the skin. The crucial factor in extending the service life of the aircraft skin lies in the effectiveness of cleaning the original paint layer. Traditional methods such as mechanical cleaning, chemical solvent cleaning, and water jet-aided cleaning have proven to be inefficient and are unable to meet industrial standards due to their tendency to degrade the surface quality of the substrate [4,5,6,7,8]. As a result, there is a lack of widespread adoption of laser cleaning technology in the industry [9]. However, laser cleaning presents a significant opportunity for advancement as an innovative cleaning technology that offers improved effectiveness and environmental benefits [10,11,12,13,14].

The primary determinants of the substrate’s surface quality and cleaning effectiveness are the laser’s properties [15,16]. Studies on the optimization of laser cleaning process parameters and the enhancement of substrate surface properties under heat effect following laser cleaning have been conducted recently. According to research by Madhuka, the most effective paint removal was accomplished at a repetitive frequency of 150 Hz with a duty cycle of 5% and a spot overlap of 50%. This study examined the impact of the laser’s operating mode on the paint removal efficacy of fiber laser [17]. Yuhang He conducted comparative tests on the corrosion resistance of cleaned metal surfaces and coatings, concluding that laser cleaning technology can improve metal surface cleanliness and corrosion resistance, reduce maintenance times, and extend service life [18]. Wenqin Li examined the cleaned sample’s surface morphology, chemical components, surface functional groups, and grain dislocation mechanism. He discovered that the surface hardness increased by 3.587% when compared to the conventionally mechanically cleaned samples, and it is thought that plastic deformation causes the substrate surface to become strain-hardened [19]. The key to the cleaning effect of laser cleaning is the connection of temperature and stress fields formed on the substrate’s surface [20]. Finite element analysis has been widely used by researchers to investigate laser–material interaction during laser cleaning [21]. Huang Dong established a two-dimensional axisymmetric finite element laser cleaning model. Through theory and experimentation, he indicated that one of the cleaning mechanisms involved in the process was thermal stress [22]. Runpeng Miao established a three-dimensional thermodynamic model of laser cleaning using ANSYS 13.0. Through simulation calculations, it was discovered that an increase in laser energy density would cause the temperature difference between the coating and substrate to increase, leading to a larger difference in thermal stresses. It is thought that the thermal stresses on the aluminum alloy substrate’s surface provide the majority of the cleaning force at the interface [23].

The primary focus of this research paper is the industrial demand for laser cleaning. A nanosecond pulsed laser was employed to conduct laser cleaning tests on an acrylic polyurethane paint layer applied to the surface of a 2024 aluminum alloy. The impact of power density, scanning speed, and pulse frequency was quantitatively analyzed using roughness and material removal rate definitions. Additionally, the evolution of the substrate’s microstructure after cleaning was examined, and a cleaning model was developed using COMSOL Multiphysics 6.1 to investigate the transient variations in the temperature and stress fields during laser cleaning.

## 2. Materials and Methods

### 2.1. Experimental Materials

The test sample utilized is an actual aircraft skin counterpart, and Table 1 displays its primary chemical compositions. The sample substrate is 2024 aluminum alloy. Before the test, the samples were cut into 20 mm × 20 mm × 3 mm pieces, polished using 400–2000 grit SiC sandpaper (GOLDSUN, Dongguan, China), and cleaned using an Ultrasonic Cleaner (AK-010S, Yu clean, Shenzhen, China) with deionized water. Acrylic polyurethane paint is frequently sprayed on the aircraft skin because of its good corrosion resistance, excellent adhesion, and other characteristics. The spray paint nozzle has a caliber of approximately 1.8 to 2.5 mm and a gun pressure of 0.3 to 0.5 MPa. Three layers are sprayed, with each layer drying at room temperature for 30 to 60 min to reach the dry surface before the second layer, with a thickness control of 70 ± 5 μm, is sprayed.

### 2.2. Experiment Conditions and Setup

**Figure 1:**
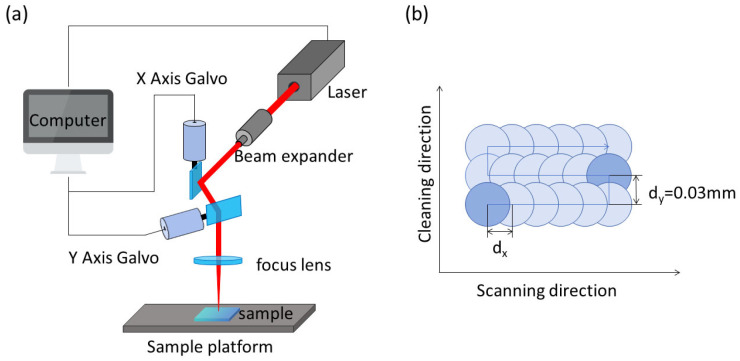
System’s schematic diagram and laser scanning path diagram. (**a**) System’s schematic diagram; (**b**) laser scanning path diagram.

Figure 1a displays the laser cleaning test system’s schematic diagram.

The laser beam is deflected with the computer-controlled scanning galvanometer system by altering the reflector’s angle in both the X and Y directions. The laser beam then travels through the focusing lens to finish cleaning the sample stage. Figure 1b depicts the laser’s scanning path. The following are the equations used for the laser energy density [24] and spot overlap rate [25]:

(1) $$\eta =\left(1-\frac{d}{2r}\right)\times 100\%$$

(2) $$F=\frac{4P}{f\pi D^{2}}$$

where *P* is the average power, *f* is the repetition frequency, *D* is the spot diameter, *r* is the spot radius, and *d* is the spot spacing. In the experiment, the *x*-direction spot spacing is defined by the formula *d_x_* = *v*/*f*, where *v* is the scanning speed, and the fixed *y*-direction spot spacing is 0.03 mm. The spot overlap rate in the *y*-direction is consequently 40%.

Table 2 displays the primary technical characteristics of the laser (MFP-20F, Max Photonics, Shenzhen, China) utilized in the experiment. The laser cleaning tests were conducted in an atmosphere devoid of any additional gas.

In order to examine how process parameters (power density, repetition frequency, and scanning speed) affect the morphology (cleaning area, depth, and roughness of the processed surface), a one-way experiment was carried out. The design scheme of the experiment is shown in Table 3, where average power is a parameter related to power density, which has 30 sets of tests set up, each of which is repeated three times.

Two indicators were used to quantify the cleaning effect: roughness and material removal rate (MMR). When the laser process settings were unable to completely clean the paint layer, the MMR—whose formula was as follows—was utilized to measure the cleaning effect:

(3) $$MRR=\left(\bar{S}\times \frac{h_{1}}{h}+\frac{h-h_{1}}{n}\right)\times 100\%$$

where $\bar{S}$ is the area ratio, *h*_1_ is the thickness of the remaining paint layer, and *h* is the total thickness of the paint layer. Following the test, laser scanning confocal microscope (LSCM-VK-250, KEYNCE, Osaka, Japan) was used to measure the roughness and 3D morphology of the cleaned surfaces; a scanning electron microscope (SEM-VEGA3, TESCAN, Brno, Czech) was used to record the morphology of the processed surfaces; an electron backscattering diffractometer (EBSD) was used to test the evolution of the substrate cross-section’s microstructure; and ImageJ.JS (ImageJ.JS (https://imjoy.io/#/), 20 March 2024) was utilized to calculate the cleaned area ratio.

### 2.3. Numerical Model

**Figure 2:**
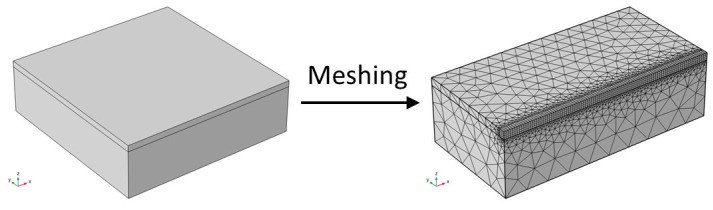
Geometric and meshing model for laser cleaning.

A two-layer heat conduction model is developed to imitate pulsed laser cleaning, assuming complete heat conduction forms between the two layers of material with no heat loss. The mesh is divided using a combination of free and mapping, and symmetry is applied to increase computing efficiency. Figure 2 displays the geometric model and meshing model.

The meshing is most detailed in the region of pulsed laser activity to enhance observation of the simultaneous imaging and analysis of the temperature, stress, and displacement fields throughout the cleaning process. The Energy Conservation Theorem and Fourier’s Theorem are satisfied by the pulsed laser cleaning paint layer’s heat conduction process. The applied laser heat source and thermal convection between the material surface and the surrounding air meet the boundary conditions. In the right-angle coordinate system, the three-dimensional heat conduction equation [26] is as follows:

(4) $$\kappa \left(\frac{\partial^{2}T}{\partial x^{2}}+\frac{\partial^{2}T}{\partial y^{2}}+\frac{\partial^{2}T}{\partial z^{2}}\right)=\rho c\frac{\partial T}{\partial t}$$

Laser heat source model [27] can be expressed as

(5) $$Q=\frac{2P}{\pi r^{2}}\left[\exp\left(-\frac{2({\left(x-x_{r}\right)}^{2}+{\left(y-y_{r}\right)}^{2})}{r^{2}}\right)\right]\frac{t}{\tau^{2}}\exp(-\frac{t}{\tau }{)}^{2}$$

Thermal convection [28] can be expressed as

(6) $$-k{\left.\frac{\partial T}{\partial n}\right|}_{\Gamma }=h\left(T_{f}-T\right)$$

where *ρ* and *c* are the material’s density and specific heat capacity, respectively; *T* is the material’s instantaneous temperature; τ is the pulse width of the laser; *t* is the thermal conduction time; κ is the thermal conduction coefficient; and *x_r_* and *y_r_* represent the distances in the *x* and *y* directions from the spot center. *n* is the normal direction, *h* is the convection coefficient, and *T_f_* is the initial temperature. An event interface has been implemented in order to achieve the dispersal of nanosecond pulsed laser in time.

The erosion rate of the solid boundary [28] is

(7) $$\nu_{a}=\frac{q_{a}}{\rho H_{s}}$$

where *v_a_* is the normal movement of the traveling velocity, *q_a_* represents the heat flux generated by ablation, and *H_s_* represents the heat of sublimation of the material.

The system has no externally applied stresses, and during the laser’s heating process, the temperature gradient created within the material causes a difference in the paint layer’s and the substrate’s coefficient of thermal expansion, which results in the generation of thermal stresses. The equation for this phenomenon is as follows:

(8) $$\rho \frac{\partial^{2}u}{\partial t^{2}}=E\frac{\partial^{2}u}{\partial z^{2}}-E\alpha \frac{\partial T}{\partial z}$$

where α is the material’s thermal expansion coefficient, *t* is the time, *u* is the displacement, and *E* is the elastic modulus. The model assumes that the substrate’s bottom is a fixed constraint, that all other barriers are free bounds, and that there is no initial displacement or stress in the system.

## 3. Results and Discussion

### 3.1. Effect of Laser Power Density on Cleaning Results

**Figure 3:**
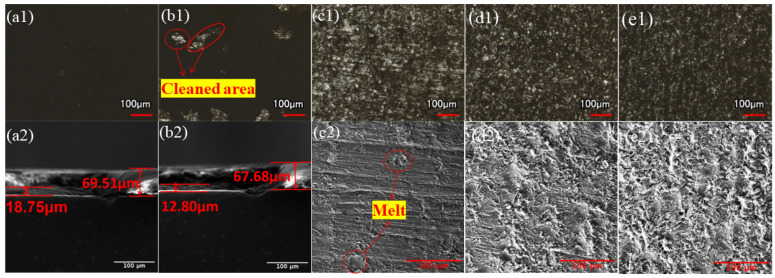
Morphology of the cleaned samples at different power densities. (**a1**,**a2**) 254.65; (**b1**,**b2**) 305.58; (**c1**,**c2**) 356.51; (**d1**,**d2**) 407.44; and (**e1**,**e2**) 458.37 MW/cm^2^.

The MMR was measured to be 73.56% at a power density of 254.65 MW/cm^2^ and 82.79% at 305.58 MW/cm^2^. The macroscopic and microscopic morphology under different power densities at a scanning speed v = 500 mm/s and repetition frequency f = 20 kHz are shown in Figure 3. The cross-section part was taken when the paint layer could not be completely cleaned, as shown in Figure 3(a2,b2). 

**Figure 4:**
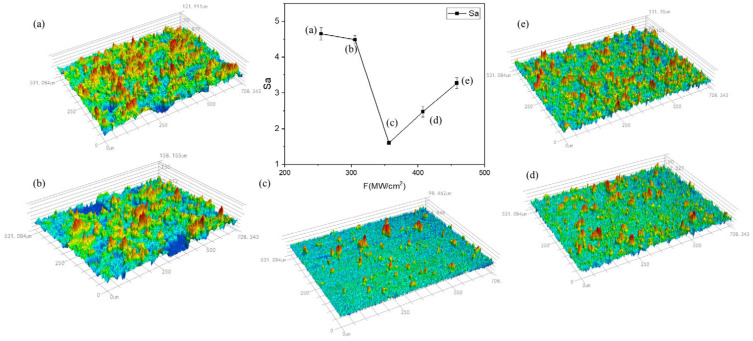
Roughness of the cleaned specimens at various power densities. (**a**) 254.65; (**b**) 305.58; (**c**) 356.51; (**d**) 407.44; and (**e**) 458.37 MW/cm^2^.

When the power density is low, the upper layer of paint is heated to the point where it melts the least, softens or melts, and is removed; close to the substrate of the paint layer, the temperature does not reach the melting temperature. As the laser power density increases, more energy is transferred to the paint layer’s surface through irradiation, improving the area of the substrate exposed to an increase in the paint layer’s MMR. As the power density reaches 356.51 MW/cm^2^, the MMR reaches 100%, and it is evident that the surface has polished traces. The paint layer absorbs a significant amount of energy from the laser’s direct irradiation, and the paint layer separates from the aluminum substrate’s surface due to thermal expansion brought on by thermal stress. As shown in Figure 3(c1,c2), the aluminum substrate slightly melts after reaching its lowest melting point, resulting in a few micro-pits created by ablative melting in the central area of the cleaning path during laser scanning. When the laser power density is increased further, the aluminum substrate’s surface temperature reaches the lowest melting point, leaving grooves from melt spattering and laser ablation. Numerous wavy honeycomb traces form, indicating that the substrate’s surface is seriously damaged. Figure 4 displays the variance in surface roughness of the cleaned specimens at various power densities.

With an increase in laser power density, there is a tendency for the cleaned specimen’s surface roughness to first decrease and subsequently increase. At a low power density, the residual paint layer covers the measured surface, resulting in a higher measured roughness. As the laser power increases, the measured roughness decreases until it reaches 1.598 μm; when the power density is reduced to 356.52 MW/cm^2^, the lowest surface roughness is found. The paint layer is completely cleaned as the power density increases; the aluminum substrate absorbs the pulsed laser energy, the surface turns molten, and the molten slurry mobility increases. However, the molten slurry moves around due to the pressure of the plasma shock wave, increasing the surface roughness. Nonetheless, it is still less than the paint layer, which has not been completely cleaned according to the roughness of the numerical value.

### 3.2. Effect of Scanning Speed on Cleaning Results

**Figure 5:**
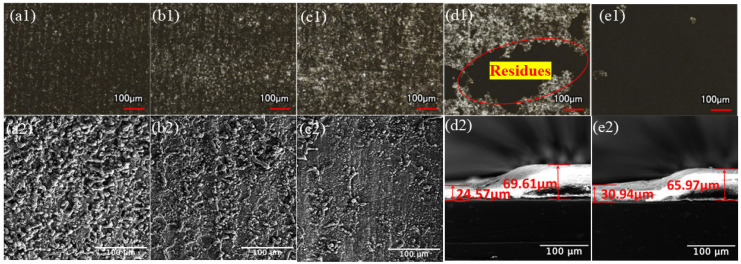
Morphology of cleaned specimens at different scanning speeds. v = (**a1**,**a2**) 300; (**b1**,**b2**) 400; (**c1**,**c2**) 500; (**d1,d2**) 600; and (**e1**,**e2**) 700 mm/s.

Figure 5 displays the macroscopic and microscopic morphology at various scanning speeds for a laser power density F = 237.67 MW/cm^2^ and repetition frequency f = 30 kHz (the cross-sectional portion was collected when the paint layer could not be fully cleaned, as shown in Figure 3(e2) and Figure 5(d2). The MMR was found to be 55.17% at 700 mm/s and 86.97% at 600 mm/s.

A 50 μm diameter Gaussian laser beam is utilized in this operation. Variations in scanning speed have an impact on the spot overlap rate, which is determined by how many times a spot is exposed. As the scanning speed reaches 300 mm/s, spot overlap rates reach up to 80%, and four times a spot is exposed. Even though the MMR is 100%, it is evident that the aluminum substrate damage blackens the cleaning of the surface of the presence of many non-uniform groove-like morphologies, as shown in Figure 5(a1,a2). Up until the speed reaches 500 mm/s, as shown in Figure 5(c1,c2), the aluminum substrate damage is reduced, the spot overlap rate gradually decreases, three times a spot is exposed, the spacing between neighboring spots grows, the pulse action time per unit area decreases, the energy absorbed by the paint layer gradually decreases, the paint layer melts, evaporation occurs, and the heat accumulation effect is weakened. Further, the scanning speed reaches the cleaning threshold, and the paint layer is completely cleaned off the substrate surface. When the scanning speed is increased further, the pulse action time per unit area is shortened, the number of exposures per point is reduced, the thermal cumulative effect is insufficient, and the paint layer gasification effect increases, but the exfoliation of paint is lessened. As a result, there is an excess of paint layer between the micro-pits when the laser is moving over the surface of the paint layer, the MMR is decreased, and the paint layer cannot be completely removed.

**Figure 6:**
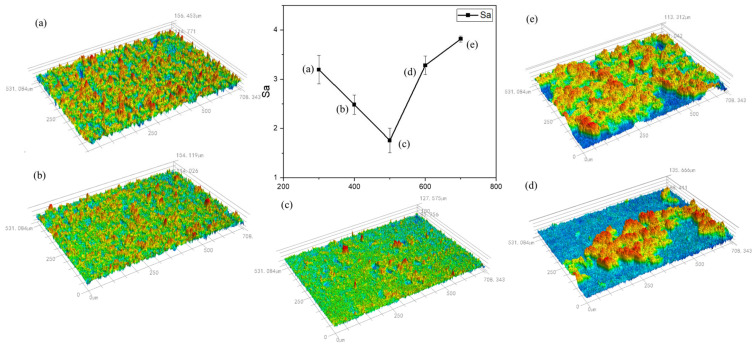
Roughness of cleaned specimens at different scanning speeds. v = (**a**) 300; (**b**) 400; (**c**) 500; (**d**) 600; and (**e**) 700 mm/s.

Figure 6 displays the variance in surface roughness of the cleaned specimens at various scanning speeds.

When the laser scanning speed increases, the cleaned specimen’s surface roughness first tends to decrease and then increases. The lowest surface roughness is reached when the scanning speed hits 500 mm/s. A high scanning speed causes the upper layer of paint to absorb laser energy, making it impossible to remove the paint layer entirely and increasing surface roughness. Conversely, a low scanning speed causes more melt to accumulate on the substrate due to thermal ablation during pulsed laser cleaning. Overall roughness is reduced at lower scanning speeds than at higher scanning speeds due to the fluidity of the molten substrate.

### 3.3. Effect of Repetition Frequency on Cleaning Results

**Figure 7:**
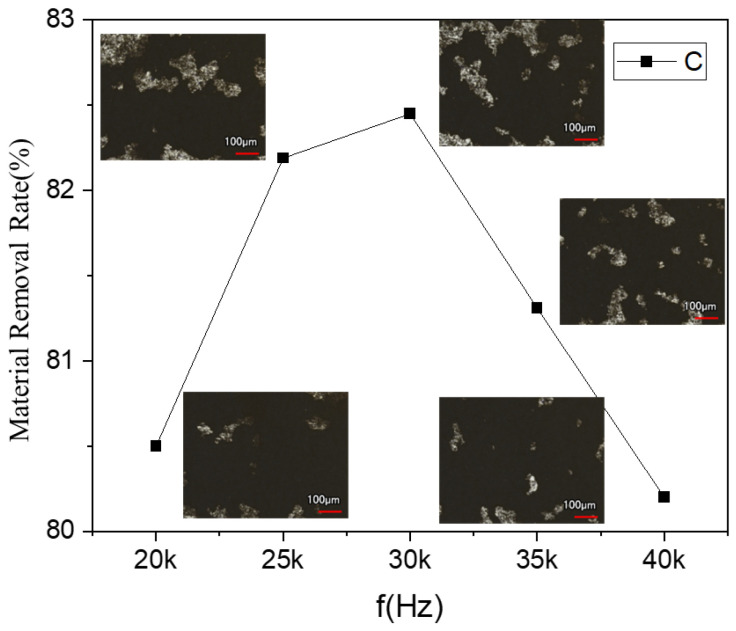
Variation of MMR for different pulse frequencies at P = 12 W.

The paint layer could not be completely cleaned at this point, reaching a thickness of 55 ± 3 μm when using a 12 W laser power and a 500 mm/s scanning speed. Figure 7 illustrates how the removal rate varied with different repetition frequencies.

Equations (1) and (2) show that as the repetition frequency is raised, the laser energy density per pulse decreases, and the spot overlap rate rises. These two factors combined have an impact on the laser cleaning results. With a repetition frequency of less than 30 kHz, the main factor is an increase in the overlap rate. The time of the pulse action in the unit area increases, and the temperature field of the pulsed laser irradiation on the paint layer’s surface is superimposed; this strengthens the heat accumulation effect and increases the MMR. With a pulse frequency of greater than 30 kHz, the main factor is an increase in the laser energy density per pulse. The paint layer absorbs less energy, and the residual increases as the MMR decreases.

**Figure 8:**
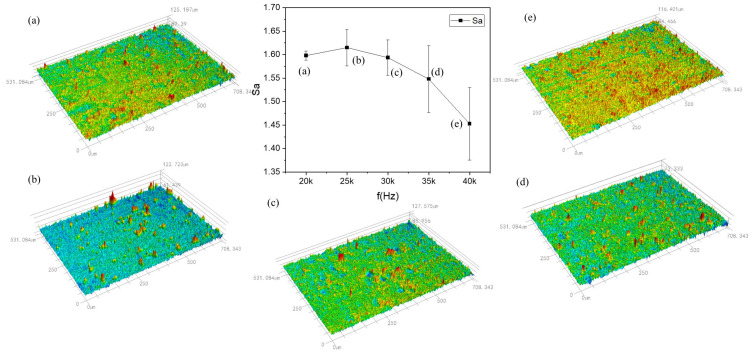
Roughness of cleaned specimens under different pulse frequencies at P = 14 W. f = (**a**) 20; (**b**) 25; (**c**) 30; (**d**) 35; and (**e**) 40 kHz.

Figure 8 displays the fluctuation of the cleaned specimens’ surface roughness under various repetition frequencies with a laser power of P = 14 W and a scanning speed of v = 500 mm/s. When the repetition frequency increases, the change in roughness increases and then drops. However, the difference in values is just 0.2 μm. This difference is because, despite the inconsistency of the repetition frequency, when we calculated the laser spot energy distribution, we found that the values of the laser spot energy distribution of different frequencies are consistent with each other. All of them are 71.3 J/cm^2^, which leads to a smaller difference in roughness, and this shows that the pulse frequency has a smaller effect on the cleaning effect. With a repetition frequency of 25 kHz, a spot overlap rate of 60%, and an energy density of 42.78 J/cm^2^, the roughness reached its maximum value of 1.615 μm at this point. As the repetition frequency increased, so did the spot overlap rate and the unit area of the time-based pulse effect, but the absorption of laser energy decreased. The value of the spot energy distribution remained unchanged, and the roughness gradually decreased, but the change in value was small until the pulse frequency exceeded 40 kHz, after which the paint layer could not be cleaned. Consequently, there is a greater impact of the laser power and scanning speed on the cleaning effect of the repetition frequency, and when the laser power and scanning speed reach a certain threshold, the repetition frequency experiences peak damage and two cleaning thresholds in the range of which the paint layer can be completely cleaned out. There is a certain value wherein the cleaning efforts to maximize the damage to the peak value of the cleaning threshold will not be able to completely clean out the paint layer outside the scope of the cleaning threshold.

### 3.4. Microstructural Evolution

**Figure 9:**
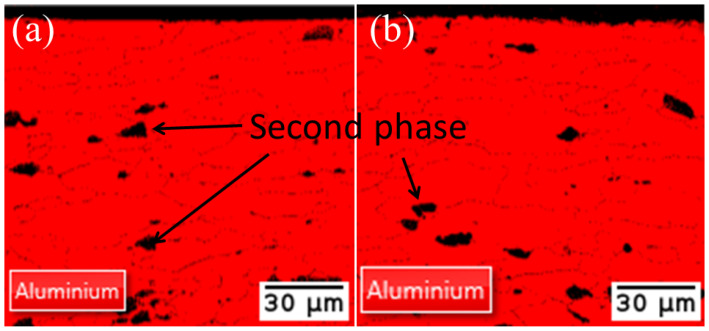
Cross-sectional phase distribution. (**a**) Original substrate; (**b**) Cleaned substrate.

The cross-sectional phase distribution diagrams are displayed in Figure 9 and are from EBSD tests conducted on the cross-sections of the original aluminum alloy plate and the cleaned substrate aluminum alloy plate, respectively. The second phase visible in the figure is represented by the black portion. This black portion represents the reinforcing phases of other elements, like Mn, Ti, and Mg. Since these phases are not discussed in this paper, they are not analyzed here. As the images show, all of the substrates are aluminum phase, and there is no phase change throughout the laser’s cleaning process since the phase of the laser-irradiated surface does not differ from the phase of the non-laser-irradiated surface.

**Figure 10:**
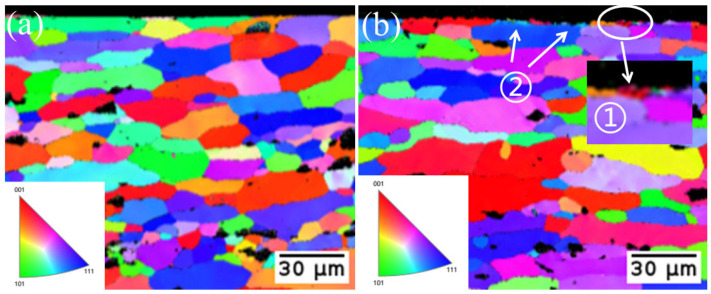
IPF. (**a**) Original substrate; (**b**) Cleaned substrate. ① Fine grains; ② Color gradient.

The cross-section’s inverse pole figure (IPF) is displayed in Figure 10. Both the original aluminum alloy substrate and the sample’s cross-section following laser cleaning lacked a substantial grain orientation, as indicated by the IPF patterns. The initial aluminum alloy substrate’s grain size is fairly uniform, as can be seen in Figure 10b. In area 1, the appearance of fine grains in some areas close to the substrate’s surface suggests that there is some grain refinement and recrystallization on the surface of the laser-irradiated substrate. A portion of the grain color gradient can be seen in Figure 10b, area 2. This gradient shows that the substrate’s surface can absorb laser energy and that some stress concentration causes some grains to accumulate dislocations. If stress is further concentrated, grain boundaries will result, and fine grain formation will begin preferentially along the grain boundaries.

**Figure 11:**
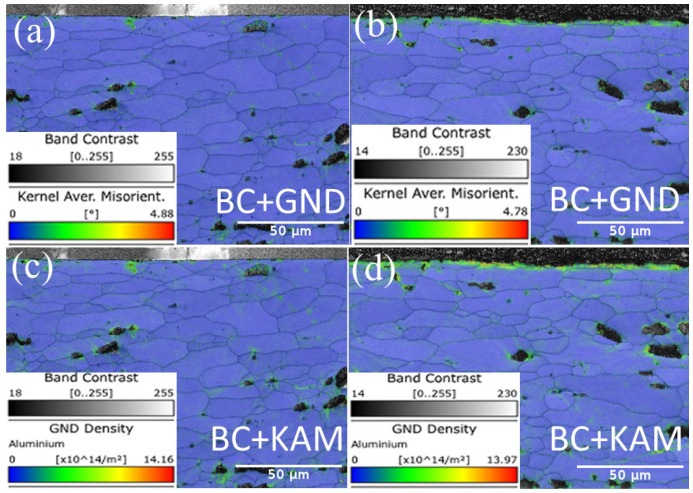
Spatial distribution of GND and KAM. (**a**,**c**) Original substrate; (**b**,**d**) Cleaned substrate.

The distribution of the kernel’s average misorientation (KAM) and geometrically necessary dislocation (GND) for the original substrate vs. the cleaned substrate cross-section is displayed in Figure 11.

The comparison shows that after laser processing, there is a strong concentration of stress on the substrate’s surface in the form of a significant localized orientation deviation. Additionally, the increase in the GND value within the grains suggests that dislocations are continuing to accumulate during the cleaning process, which may lead to the formation of fine grains. Conversely, a greater GND value in the laser action’s surface area indicates that the dislocations produced there are more concentrated and will be absorbed by new grains, in line with the recrystallization concept. Since the laser is driving the microstructure evolution, the laser-cleaned substrates’ greater GND values than the pristine sample’ suggest this.

The KAM plots in Figure 11c,d make it clear that after the laser action, the stress value increased in comparison to the original sample and that a significant amount of stress had accumulated inside the grains on the substrate surface, which can be used to drive the formation of recrystallization. The steady shift in grain color and the finer grain development in the IPF plots are validated by the distribution of GND and KAM.

### 3.5. Simulation Result

**Figure 12:**
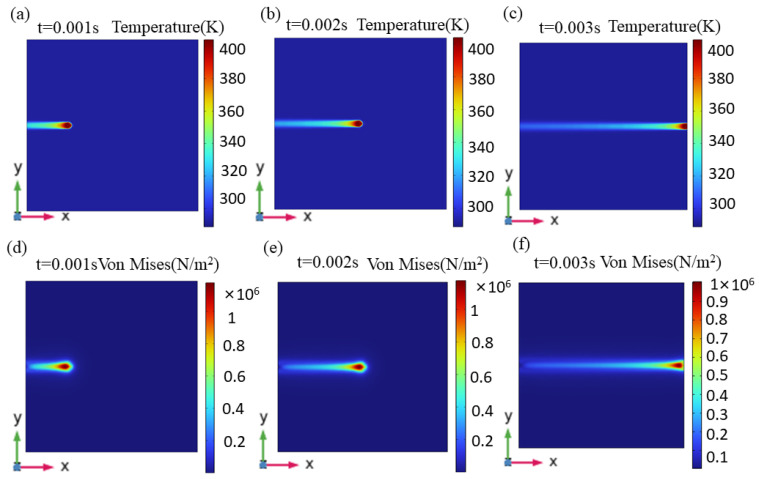
Temperature field and stress field distributions at different moments. t = (**a**,**d**) 0.001; (**b**,**e**) 0.002; and (**c**,**f**) 0.004 s.

Figure 12 displays the temperature field distribution and stress field distribution at various points in the laser’s cleaning procedure. The temperature field distribution during the laser’s cleaning procedure follows a Gaussian distribution, with the highest temperature in the center of the spot and lower temperatures on the borders. High-speed movement of a single pulsed laser linked to a specific overlap rate, causing the neighboring laser action areas to overlap partially, superposing laser energy and acting on the paint layer’s surface. Following the paint layer area’s pulsed laser scanning, the temperature rises quickly, and a large temperature gradient forms around the spot because the material being heated by the pulsed laser has hot and cold characteristics. In the residual scanning path, the temperature difference increases as the pulse has not yet cooled down.

The paint layer’s convection with the surrounding temperature followed laser scanning, producing the highest stress values close to the center of the adjacent spot. This distribution of stress fields is similar to that of the temperature field. The spot travels along the cleaning path, the substrate temperature rises, and the heat-affected zone moves over time. However, the paint layer’s surface around the spot absorbs less energy, and the temperature rises more slowly than the spot center temperature, and a steep temperature difference forms. After the cleaning is finished, the surface thermal stress starts to decrease as a result of the dissipation of thermal energy.

**Figure 13:**
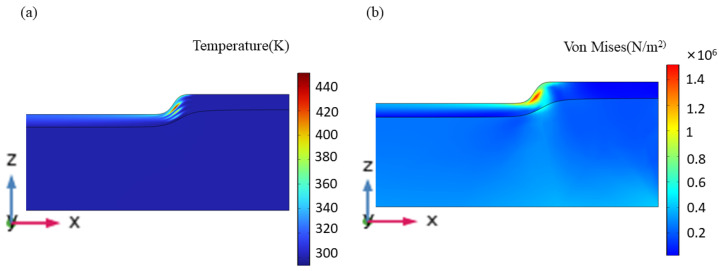
Ablation morphology after laser cleaning. (**a**) Temperature field; (**b**) stress field.

Figure 13 displays the temperature field, stress field, and ablation morphology following laser cleaning.

Due to the paint layer’s small absorption coefficient and weak laser penetration ability, the temperature difference close to the substrate’s surface varies greatly in depth and direction. A portion of the paint layer’s temperature is below the threshold value of vaporization, resulting in the removal of a large portion of the paint layer while leaving some paint on the surface. Rapid temperature changes cause the stress on the paint layer’s surface to decrease quickly, but they also cause heat to be superimposed on the aluminum alloy substrate’s surface over time and create a stress differential between the paint layer’s and the aluminum alloy’s contact surfaces. This difference causes the paint layer that is still on the substrate’s surface to expand thermally, causing it to separate from the substrate and become completely cleaned. This suggests that during the cleaning process, thermal vibration and ablation mechanisms coexist.

## 4. Conclusions

In this research study, a nanosecond pulsed laser was used to remove acrylic polyurethane paint from 2024 aluminum alloy. This technology can be applied to cleaning procedures in the industrial field. This experiment revealed the following:

1. Without causing harm to the substrate, the laser may thoroughly clean the paint layer. With a wavelength of 1064 nm, a pulse width of 100 ns, a spot diameter of d = 50 μm, a scanning speed of v = 500 mm/s, a power density of 178.25 W/cm^2^, and a pulse frequency of f = 40 kHz, the ideal parameters for cleaning a 70 μm thick paint layer are 100% paint layer removal and the lowest possible roughness.
2. A cleaning effect with greater impact is affected by the laser power and scanning speed, while a smaller impact is cleaning via pulse frequency. When the scanning speed and laser power are equal, the pulse frequency has two cleaning thresholds: one that can completely remove the paint layer within the cleaning threshold range, and another that is peak damage. The paint layer will not be entirely removed if the cleaning threshold range is exceeded.
3. Due to laser irradiation, the substrate surface’s dislocation accumulation causes a concentration of stress that causes the emergence of grain refinement. Both the original substrate and the substrate cleaned with a laser show no obvious grain orientation, and there is no phase transition after cleaning.

## Figures and Tables

**Table 1:** The chemical compositions of the 2024 aluminum alloy.

Cu	Mn	Mg	Cr	Si	Zn	Al
3.8~4.9	0.3~1.0	1.1~1.8	0.10	0.5	0.25	Bal.

**Table 2:** Main technical parameters of the laser.

Wavelength (nm)	Power (W)	Pulse Width (ns)	Frequency (kHz)	Scan Speed (cm·s^−1^)	Spot Diameter (μm)
1064	<20	100	20–80	<20,000	50

**Table 3:** The design scheme of the experimental.

Number	Power (W)	Frequency (kHz)	Scan Speed (cm·s^−1^)
1~5	10, 12, 14, 16, 18	20	500
6~10	10, 12, 14, 16, 18	25	500
11~15	10, 12, 14, 16, 18	30	500
16~20	10, 12, 14, 16, 18	35	500
21~25	10, 12, 14, 16, 18	40	300, 400, 500, 600, 700
26~30	14	30	300, 400, 500, 600, 700

## Data Availability

The original contributions presented in the study are included in the article; further inquiries can be directed to the corresponding author.
